# Internal mucosal resection and LLC suspension: a new method to establish a stable tip rotation angle and reduce the nasal bridge length

**DOI:** 10.55730/1300-0144.5808

**Published:** 2024-01-05

**Authors:** Mehmet DADACI, Moath ZUHOUR, Bilsev İNCE, Zeynep ALTUNTAŞ

**Affiliations:** 1 Department of Plastic, Reconstructive and Aesthetic Surgery, Necmettin Erbakan University Meram Faculty ofMedicine, Konya, Turkiye; 2 Department of Plastic, Reconstructive and Aesthetic Surgery, Private Hospital of Büyükşehir, Konya, Turkiye; 3 Department of Plastic, Reconstructive and Aesthetic Surgery, Private Clinic, İzmir, Turkiye; 4 Department of Plastic, Reconstructive and Aesthetic Surgery, Hospital of Farabi, Konya, Turkiye

**Keywords:** Cartilage suspension, long nose, mucosal resection, rhinoplasty, tip rotation

## Abstract

**Background/aim:**

Herein, we describe a new technique to obtain both the appropriate degree of rotation angle and the ideal nasal bridge length. The aim of this study is to investigate the long-term results of this new technique with regard to these two variables.

**Materials and methods:**

A total of 76 (27 males, 49 females) patients were operated in accordance with the presented technique. Internal caudal mucosal excision and lower lateral cartilage (LLC) suspension were applied to all the patients included in this prospective study. Preoperative, immediate postoperative, and postoperative 1^st^-year photographs were taken. NOSE scores were obtained in the postoperative 1^st^ year.

**Results:**

The mean nasolabial angle values of the patients preoperatively, at the end of the surgery (immediate postoperative), and at the end of the first year were 94.13° ± 5.1, 113.1° ± 5.3, and 109.6° ± 5.2, respectively. The patients had an average gain of 19° at the nasolabial angle at the end of the surgery and experienced a 3.5° (3.1%) loss at the end of the first year. For the nasal bridge length (n-prn) values; the preoperative, immediate postoperative, and first year mean values were 5.1 ± 0.55 cm, 3.98 ± 0.41 cm, and 4.29 ± 0.39 cm, respectively. The noses of the patients were shortened by 1.11 cm on average at the end of the surgery.

**Conclusion:**

Internal caudal mucosal resection with a suspension of the LLC to the caudal edge of the upper lateral cartilages (ULC) offers a reliable method to control the nasal tip rotation and shorten the long noses. This technique’s effect is more obvious in long noses compared to the short ones.

## 1. Introduction

Rhinoplasty is considered to be one of the most complex aesthetic surgeries, in which many variables such as nasal tip rotation angle, tip projection, and nasal length play a role. Until now, many techniques have been defined to achieve the ideal nasal measurements. Since different deformities are encountered in different noses, it is very difficult to apply one “standard technique” to all the patients.

The ideal tip rotation angle is defined to be 90°–95° for men and 95°–115° for women [1,2]. Although the nasolabial angle is the most commonly used method for measuring this angle, there are many other methods, such as the columellar-facial angle in relation to the facial plan, the columellar-facial angle in relation to the line drawn perpendicular to the Frankfort line, or the nostril axis angle in relation to the line drawn perpendicular to the Frankfort line [3–5]. To adjust the appropriate tip rotation angle, Webster was the first to describe the lateral crural flap technique in 1975 [6]. After that many techniques such as lateral crus overlay, lateral crus steal, and tongue-in-groove have been described to enhance the stability and reliability of this angle [7].

The ideal nasal length should be 2/3 of the vertical length of the midface. Likewise, it is recommended that the tip projection be 2/3 of the vertical length of the nose and 2–3 mm higher than the dorsum to make it look natural [8]. The measurement, called ‘the nasal bridge length’, expresses the distance between the Nasion and Pronasale (n-prn). The normal value of this length varies between 4.5–5 cm [9]. Considering the anatomical basis, the pathology of long noses can be examined in two groups. The main pathology in type 1 long noses is the long septal cartilage. In this group, although the complex of the alar cartilages has a normal ligamentous connection with the anterior septal angle, it is located inferiorly due to the long septal and upper cartilages [10]. In type 2 long noses, there are abnormal aponeurotic connections between the alar cartilages and the septal angle. This anomaly causes the alar cartilages to tend down easily [11]. The mainly used techniques for shortening the long noses are excision of the caudal ends of the upper lateral cartilages, reduction of the anterior septal angle and modification of the caudal septal cartilage [12,13]. If the pathology originates from the alar cartilages independently of the septum, this problem can be solved by fixing the tip to the anterior septal angle [11].

In this study, we aimed to investigate the long-term results of the newly described technique on nasal bridge length and tip rotation angle.

## 2. Methods

### 2.1. Study design

A total of 76 patients who underwent rhinoplasty surgery between March 2019 and May 2020, were included in this prospective study. Written consent was provided, by which the patients agreed to the use and analysis of their data. The study was planned and conducted in accordance with the World Medical Organization’s Declaration of Helsinki. The ethics committee of clinical trials with nonpharmaceutical products and medical devices of our institution (protocol number 2019/2231) approved the study. All operations were performed by the same surgeon.

Patients were followed for at least 1 year when their late postoperative photographs were taken and NOSE scores were obtained. Photographs were analyzed by Digimizer Image Analysis Software (version 5.4.1. MedCalc Software Ltd., Ostend, Belgium) to calculate the tip rotation angle and nasal bridge length (n-prn). The angle of rotation was determined by measuring the nostril axis angle in relation to a line perpendicular to Frankfort horizontal line. The postoperative 1^st^-year tip rotation angle and nasal bridge length (n-prn) values were compared to those of preoperative and immediate postoperative period.

### 2.2. Surgical technique

Surgery was initiated in accordance with the open approach by columellar step and infra-cartilagenus rim incisions. Following elevating the skin envelop, septal cartilage was exposed at subperichondrial plane. When septal deviation was present; deviated parts were excised to bring the septal cartilage to the midline. In most cases, 3–5 mm caudal septal excision was enough to shorten the long septum. When necessary, septal cartilage was fixed to the anterior nasal spine. After bilateral spreader flaps were created, the reduction of the nasal hump began with the excision of the superior septal cartilage. The amount of excision varied according to the size of the hump.

**Figure 1:**
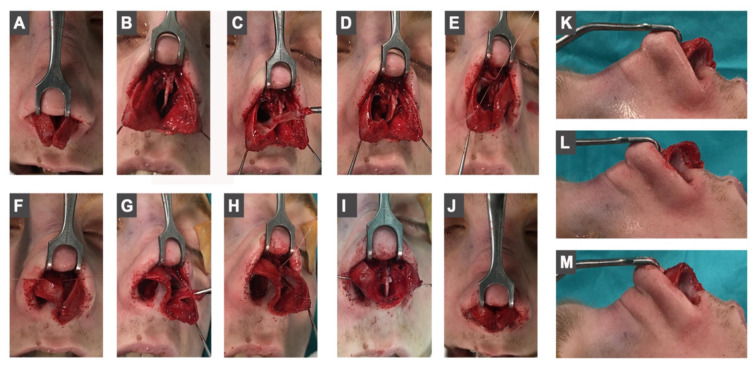
Intraoperative photographs of the described technique, A–B: preoperative view of the LLC’s, C: bitriangular mucosa excision is done by the same incision, D: view of the mucosal excision extending posterocaudally in the direction of ANS, E: a 4-0 rapid absorbable suture is passing from beneath the domal segment to the caudal edge of the ULC, F: the position and shape of the LLC after fixing the suspension suture, G–H: the same procedure is applied to the left side of the LLC, I: the area between ULC’s and LLC’s after the excision and suspension process, J: the shape and position of the LLC’s after bilateral excision and suspension, K: lateral view of the LLC before the procedure, L: lateral view of the right LLC after the excision and suspension procedure, M: lateral view of the LLC’s after bilateral procedure is applied

The bony hump was lowered with the help of osteotomy and/or rasp, then it was followed by lateral and transverse osteotomies. After suturing the spreader flaps to the septal cartilage, cephalic trimming of the lateral crura and caudal trimming of the ULC were applied. Later, the lateral crura were retracted infero-laterally with the help of a hook and bitriangular mucoperichondrial excision was performed (Figure 1A–1D). The apex of the first triangle points toward the caudal end of the ULC, while the apex of the second triangle is designed to be in the direction of the anterior nasal spine (ANS). The first triangle is necessary for creating the desired shortening and rotation effect. However, the second triangle is done to eliminate the folded mucosa which results from the shortening process. Bitriangular excision is done at the same time with the same incision. The base-width of these triangles is designed according to the desired degree of rotation and nasal length. Care must be taken to leave a safety zone beneath the domal segment in order not to cause an over-rotated or very short nose. Then, a suspension suture was passed through the mucosa underneath the domal segment to be connected with the caudal edge of the ULC where the spreader flaps end (Figures 1E–1J).

At the end of this procedure, 5 basic events occur:

-Nasal bridge shortening (n-prn).
-Superior elevation of the columella.
-Cephalic rotation of the alar domes (Figures 1K–1M).
- Caudal rotation of the lateral crura
-Disappearance of the mucosal folds at the medial wall of the nostrils which occurs due to the shortening process.

After placing tip creating and interdomal sutures, the skin and mucosa incisions are sutured. Suturing the mucosa along the columellar-septal junction line (incision line resulting from mucosal triangle excision) provides support for this technique. After the standard photographs are taken, the operation is terminated by placing bilateral silicone pads and nasal cast. No septocolumellar suture, columellar strut, cap/tip grafts, or tongue-in-groove was used in the surgery. To achieve lower intraoperative pain response, anesthesiologists were asked to increase the Remifentanil infusion rate before the most painful procedures (periost elevation, osteotomy and lower turbinate lateralization) [14].

### 2.3. Statistical analysis

Data were analyzed with SPSS 24.0 (IBM, Armonk, NY) software. The difference in the degree of nasal tip rotation and nasal length between preoperative, immediate postoperative, and postoperative 1^st^ years were analyzed using the t-test for normally distributed data and Mann–Whitney U test for abnormally distributed data. The normality of the distribution was checked using the Kolmogorov–Smirnov test. To investigate the correlation between preoperative and postoperative results, a linear regression model was used.

## 3. Results

A total of 76 patients were included in this prospective study. The mean age of the patients was 26.9 ± 5.6 (19–43). 49 (64.4%) patients were female and 27 (35.5%) were male. Standard photographs were taken preoperatively, at the end of the procedure (immediate postoperative), and at the end of the 1^st^ year.

The mean nasolabial angle values of the patients preoperatively, immediate postoperatively, and at the end of the first year were 94.13° ± 5.1, 113.1° ± 5.3, and 109.6° ± 5.2, respectively. Our patients had an average gain of 19° in the nasolabial angle at the end of the surgery and experienced a 3.5° (3.1%) loss at the end of the first year. For the nasal bridge length values; the mean preoperative, immediate postoperative and first year values were 5.1 ± 0.55 cm, 3.98 ± 0.41 cm, and 4.29 ± 0.39 cm, respectively. The noses of the patients shortened by 1.1 cm on average at the end of the surgery. Considering the values at the end of the first year, the noses of the patients showed a regression of 0.3 cm (7.7%). Eventually, they were shortened by 0.81 cm (Table).

When patients were separated according to sex; male patients had a preoperative 90.9° ± 3.5°of rotation. This degree increased to 111.3° ± 6.3° at immediate postoperative and decreased to 107° ± 5.9° at the end of the first year. For female patients, these values were 95.8° ± 5.1°, 114.1° ± 4.5°, and 110.8° ± 4.4°, respectively. The male patients had an increased preoperative nasal bridge length of 5.3 ± 0.44 cm compared to female patients who had a preoperative value of 4.95 ± 0.56 cm. This required a higher shortening percentage for male patients compared with females, with values of 23%, 21%, respectively (Table).

**Figure 2:**
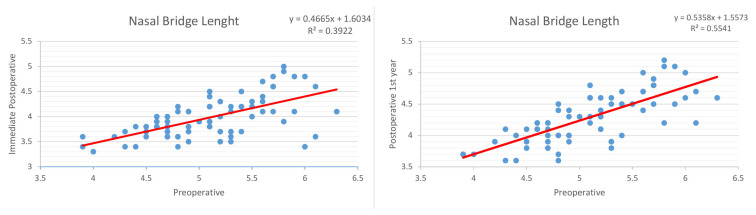
Linear regression model between preoperative and immediate postoperative, and between preoperative and postoperative 1^st^ -year nasal bridge length measurements.

The linear regression model showed a strong correlation between preoperative and immediate postoperative nasal bridge measurements with a correlation value (R) of 0.62. It also showed a strong correlation between preoperative and postoperative 1^st^-year measurements, with a correlation value (R) of 0.74 (Figure 2).

**Figure 3:**
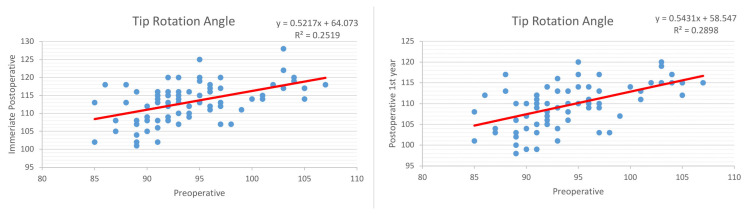
Linear regression model between preoperative and immediate postoperative, and between preoperative and postoperative 1^st^-year tip rotation angle measurements.

For rotation angle, these values were 0.501 and 0.53, respectively, and showed a moderate correlation (Figure 3). Considering these values, it can be estimated that for every 1° rotation angle we increase during the surgery, there will be a loss of 0.18° at the end of the first year, as so there will be a 0.27 cm relengthening for every 1 cm we shorten.

A statically significant difference was found at all comparisons between preoperative and immediate postoperative, and between preoperative and postoperative 1^st^ year angle and length measurements (p < .005). A similar difference was also found between the immediate postoperative and the postoperative 1^st^ year measurements (p < .005).

The patients were followed up for a minimum of 1 year. A total of 6 (7.8%) patients were reoperated. Of these, 2 were operated for residual hump, 1 for asymmetric osteotomy, and 3 for nasal obstruction due to long LLCs. We did not encounter any complications of overrotated or deprojected tips or very shortened noses in any of our patients. The mean postoperative 1^st^-year NOSE score was 12.25 ± 9.1.

## 4. Discussion

Most of the patients undergoing rhinoplasty surgery have deformities regarding the nasal tip. Droopy tip is a common complaint among these patients. However, droopy tips can also lead to a long nose appearance [15]. Tripod theory, which is one of the oldest basic theories on this subject, has emphasized that the length of the medial and lateral crura should be changed to increase the tip rotation angle. The theory argues that these cartilages have important effects on the dynamics of the nasal floor [16]. Since then, many grafting and suturing techniques have been developed. Among the grafting techniques, the most well-known is the columellar strut technique. In this technique, it is aimed to increase the tip rotation by increasing the length of the inferior leg of the tripod. Thus, a cartilaginous graft is placed between both medial crus along the caudal septum [17].

In a study by Şirinoğlu on 44 patients, he investigated the effects of columellar strut and septo-columellar sutures on nasal tip projection and rotation. The mean preoperative, postoperative 1st month and 1st year values of patients who received only septo-columellar suture were 98.5°, 111.9°, and 107.3°, respectively, while the values of the group who received only columellar strut were 104.6°, 116.2°, and 111.6°, respectively. Patients who received only septo-columellar sutures had a gain of 13.4° at the 1st month and lost 4.1% of this value at the end of the 1st year. In the patients who received only columellar strut, an increase of 11.6° was observed at the 1st month, and at the end of the 1st year, they lost 4% of this value. There was no statistical difference between the two groups [18]. The tongue-in-groove technique is another technique based on suturing the medial crus to the caudal septal cartilage to increase tip rotation. Antunes et al. investigated the long-term effects of this technique on the tip rotation. The mean nasolabial angle values of the patients at preoperative, operating room and at the end of the first year were found to be 93.95°, 114.47°, and 106.55°, respectively. Although the increase in the mean angle value was 20.52° at the end of the surgery, 6.8% of this angle was lost at the end of the first year [19].

**Figure 4:**
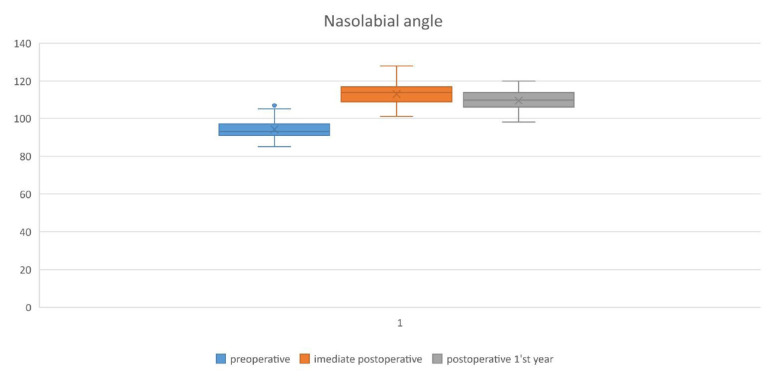
Mean values of rotation angle preoperatively, immediate postoperatively, and at postoperative 1^st^-year.

The values of the patients included in our study in preoperative, operating room, and at the end of the 1st year were 94.13° ± 5.1, 113.1° ± 5.3, and 109.6° ± 5.2, respectively; the amount of angle increase at the end of the surgery was 19°, and only 3.1% loss was experienced at the end of the first year (Figure 4).

**Figure 5:**
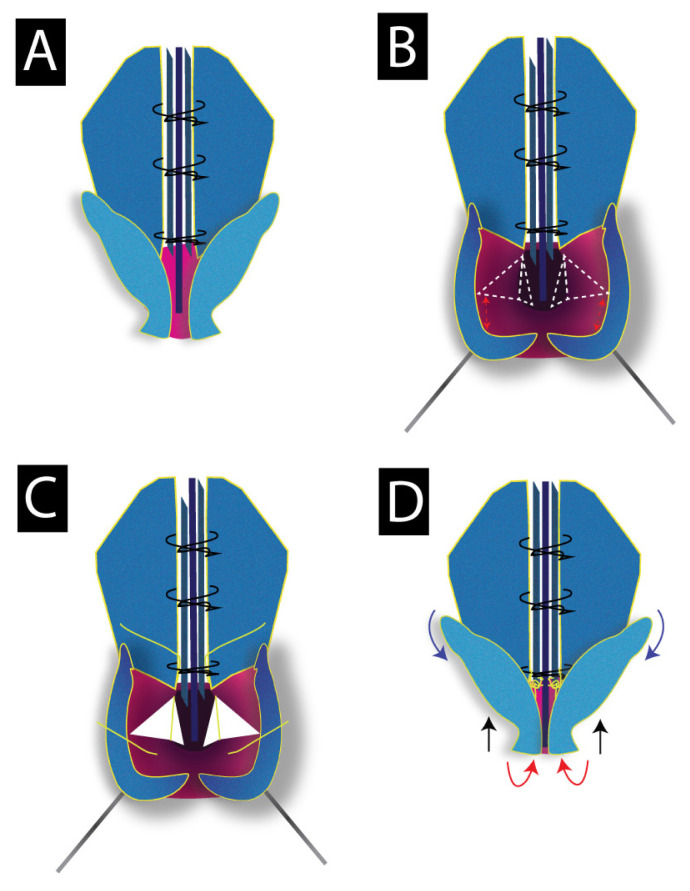
Illustration demonstrating the described technique, A: the preoperative shape of the LLC’s, B: dotted lines describe the design of the bitriangular mucoperichondrial excision, red arrows indicate an area of at least 4-mm to be left intact, C: suspension suture is passed from beneath the domal segment to the caudal edge of the ULC’s. Note that the suspension suture is passed beneath the most caudal suture of the spreader flaps to increase its reliability, D: view of LLC’s after the excision and suspension procedure, the red arrow indicates the domal segment’s cephalic rotation, blue arrow indicates the LLC’s caudal rotation, black arrows indicates the shortening effect with the columella elevation.

In this study, although cartilage grafts or septal fixation sutures were not used, we observed a fairly constant rotation angle. In other words, we can estimate that for every 1° we increase during the surgery, we will lose 0.18° at the end of the first year. When these rates are compared with the aforementioned studies and the information in the literature, we can state that our technique allows more rotations and causes less loss in the long term. Although this seems as an advantage, we would like to emphasize that exaggerated overcorrection should be avoided in order not to cause an overrotated tip. Similarly, an area of at least 4 mm beneath the domal segment should be left intact to avoid deprojection or excessive rotation (Figures 5A–5D).

We believe that the pathologies forming long nose deformity do not exist as independent events, but are found together at different impact ratios, for example in type 1 long nose, the main problem will be the long septal cartilage, but the patient will also have a long ULC and a long mucosa. The technique presented in this study is suitable to handle both type 1 and type 2 long noses at the same time, with the opportunity to modify according to the existing pathology. While septal and ULC caudal resection handles the main pathology in type 1, mucosal excision and LLC suspension establish a new relationship between LLC and ULC by eliminating the excessive space between these two cartilages. In type 1, the septal and ULC excisions are made at the same ratio as the mucosal excision. In type 2 more mucosal resection is required than cartilage excision.

Since we advocate the concept of “reduction rhinoplasty” in long noses, we are considering shortening all structures, even the mucosa. Whereas, all techniques described so far to treat long noses have focused on the LLC, ULC, or septal cartilages. Although some authors have performed caudal mucosal resection, this resection was planned to elevate the columella by using an external approach [20]. We could not encounter any study in the literature performing mucosal resection in internal fashion as we described. Considering the findings of our study, we show that mucosal resection and LLC suspension technique are reliable in modifying tip rotation and nasal bridge length. The low NOSE score obtained in the first year indicates that the technique has no negative effect on the breathing function. Contrarily, we believe that eliminating the mucosa folds at the medial wall of the external nasal valve contributed to this low score. However, septoplasty and spreader flaps were applied to all of the patients included in this study [21,22].

**Figure 6:**
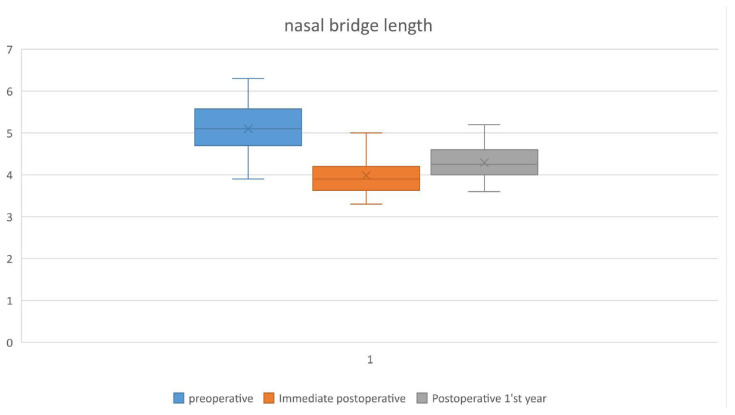
Mean values of nasal bridge length preoperatively, immediate postoperatively, and at postoperative 1^st^-year.

The mean preoperative, immediate postoperative, and postoperative 1^st^ year nasal length (n-prn) measurements were as follows, 5.1, 3.98, and 4.29 cm, respectively. The noses of our patients were shortened by 1.12 cm on average at the end of the surgery, but at the end of the first year, they regained 7.7% of this shortening. As a result, the noses of the patients shortened by 0.81 cm at the end of the first year compared to the preoperative measurements (Figure 6). The amount of this shortening increased as the nasal length increased.

The regression module data showed a stronger relationship between the postoperative and preoperative length values compared to the rotation angle values. This means that the preoperative nasal length affected the postoperative result more directly. In other words, the effect of this technique becomes more obvious in patients with longer noses. The longer the nose, the more it should be shortened, and the more it is prone to relengthen. Since the tip rotation angle shows a more stable course, it is not affected by the preoperative value as much as the nose length.

As the ULC cartilages go cephalic, they diverge from the midline toward the ipsilateral lateral canthus at an angle of 45°. In ULC cephalic malposition, this angle is less than 30° [23]. Basically, two techniques are used to correct the cephalic malposition of the ULC; transposition and lateral crural overlay [24]. In the technique we have described, some caudal rotation of the lateral crus is expected due to the cephalic rotation of the medial crus of the LLC. However, this amount of rotation may be insufficient in very long noses. Nasal obstruction was observed in three patients who underwent revision because of herniation of the long lateral crus at the nostril’s lateral walls. The patients were reoperated under local anesthesia, a 2–3 mm excision was applied to the lateral crura and then they were placed in the newly created pocket. We observed that the complaints of the patients improved at the follow-ups. It should be kept in mind that after applying the LLC suspension technique, it may be necessary to apply overlay or transposition, particularly in long and cephalically oriented lateral crus.

**Figure 7:**
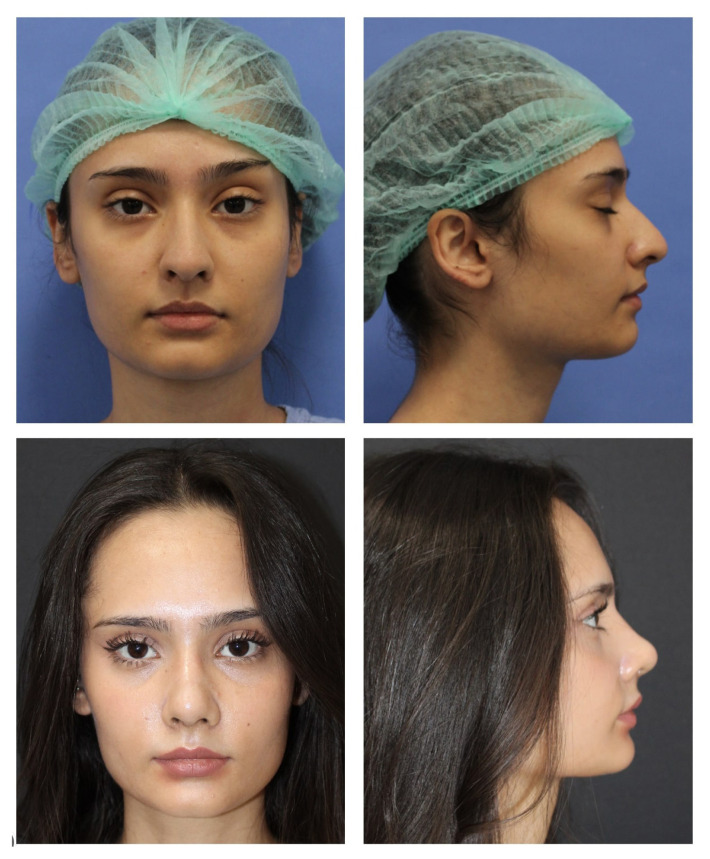
Preoperative and postoperative 1^st^-year photographs of a female patient.

**Figure 8:**
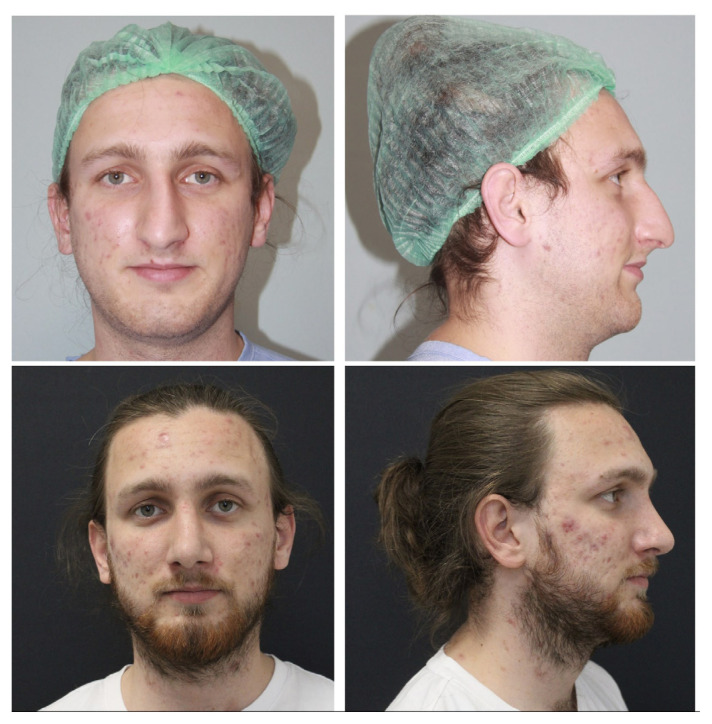
Preoperative and postoperative 1^st^-year photographs of a male patient.

Considering our findings, we believe that the technique described in this study provides a reliable method for obtaining a stable tip rotation angle in long noses. However, this technique has a learning curve. We would like to emphasize that in the preoperative period, good observation of the existing deformities, calculation of the desired degree of rotation, and the nasal bridge length are important in planning the amount of caudal septum and mucosa to be excised (Figures 7,8).

Among the limitations of our study; the absence of a comparison group and the lack of a preoperative NOSE survey can be counted.

## 5. Conclusion

The described technique offers a reliable method for establishing a stable tip rotation angle without the need for excessive overcorrection. The technique is also unique in shortening long noses in both types 1 and 2. The technique is suitable for a wide range of noses, but naturally, the shortening effect is more obvious in long noses. Although we recommend a controlled overcorrection while shortening the nasal bridge, we do not recommend it for the tip rotation angle. More studies are required to test the effect of caudal mucosal resection on breathing.

## Figures and Tables

**Table:** Data of the patients.

Parameter	All patients (n = 76)	Male patients (n = 27)	Female patients (n = 49)
**Age**	26.9 ± 5.6	30 ± 6	25.2 ± 4.7
**Preoperative angle**	94.13° ± 5.1	90.92° ± 3.5	95.89° ± 5.1
**Immediate Postoperative angle**	113.1° ± 5.3	111.3° ± 6.3	114.18° ± 4.57
**Angle change rate** #	+20%	+22%	+19%
**Postoperative 1** **^st^** ** year angle**	109.6° ± 5.2	107° ± 5.9	110.81° ± 4.47
**Angle change rate** *	−3.1%	−3.3%	−2.9%
**Preoperative n-prn length (cm)**	5.1 ± 0.55	5.3 ± 0.44	4.95 ± 0.56
**Immediate postoperative n-prn length (cm)**	3.98 ± 0.41	4.1 ± 0.36	3.9 ± 0.41
**Length change rate (cm)** #	−22%	−23%	−21%
**Postoperative 1** **^st^** ** year n-prn length (cm)**	4.29 ± 0.39	4.44 ± 0.31	4.20 ± 0.41
**Length change rate (cm)** *	+7.7%	+7.8%	+7.6%

# Change rates are calculated as the difference as a percentage compared to the preoperative value.

* Change rates are calculated as the difference as a percentage compared to the immediate postoperative value.
